# Nickel-phytic acid hybrid for highly efficient electrocatalytic upgrading of HMF

**DOI:** 10.3389/fchem.2023.1199921

**Published:** 2023-05-18

**Authors:** Shuyi Liu, Xue Yuan, Xin Huang, Yu Huang, Chen Sun, Kun Qian, Wenjie Zhang

**Affiliations:** ^1^ School of Water Resources and Environment, China University of Geosciences, Beijing, China; ^2^ School of Science, China University of Geosciences, Beijing, China

**Keywords:** biomass utilization, electrocatalytic oxidation, 5-hydroxymethylfurfural, 2, 5-furandicarboxylic acid, phytic acid

## Abstract

Electrocatalytic upgrading of 5-hydroxymethylfurfural (HMF) provides a promising way to obtain both high-value-added biomass-derived chemicals and clean energy. However, development of efficient electrocatalysts for oxidizing HMF with depressed side reactions remains a challenge. Herein, we report a nickel-phytic acid hybrid (Ni-PA) using natural phytic acid as building block for highly efficient electrocatalytic oxidation of HMF to 2, 5-furandicarboxylic acid (FDCA). Due to the coordination of nickel ion and phosphate groups of phytic acid molecule, high selectivity and yield of FDCA were achieved at 1.6 V vs. RHE. Besides, Ni-PA has a higher electrochemical surface area and lower charge-transfer resistance than Cu/Fe-PA, which significantly promotes the oxidation of HMF to FDCA. This work demonstrates the potential of metal-phytic acid hybrids as effective electrocatalysts for biomass valorization.

## 1 Introduction

The increasingly serious environmental pollution and the depletion of fossil fuels have prompted great efforts to develop environment-friendly conversion and clean energy storage technologies (Xie et al., 2018; El-Emam and Özcan, 2019; Mohammed-Ibrahim and Sun, 2019; Haldar and Purkait, 2021). The utilization of abundant and renewable biomass into fine chemicals through direct use of renewable electricity at ambient temperatures and pressures is a significant development direction and has attracted considerable interest (Zhou et al., 2021a; Zheng et al., 2022).5-hydroxymethylfurfural (HMF) is one of the most important biomass platform molecules, which could serve as essential bridge connecting resource to produce industrial chemicals (Lăcătuș et al., 2018; Kong et al., 2020), textiles (Dhivya et al., 2019), and pharmaceutical intermediates (Shao et al., 2022). Over past decades, electrocatalytic oxidation of HMF has attracted much attention due to its obvious advantages of ambient and easily controlled condition compared with aerobic oxidation (Chen C. et al., 2021; Zhao et al., 2021). However, the electrochemical oxidation of HMF still remains a challenge that the competition between water oxidation and HMF oxidation in aqueous media might lead to the decrease of selectivity and yield of FDCA. Thus, many efforts have been made on design of efficient electrodes to improve the HMF conversion efficiency and selectivity of FDCA (Mankar et al., 2019; Pang et al., 2022; Jiang et al., 2023).

In regard of the high cost and undesirable catalytic performance of noble metals, transition metal compounds (Ni, Fe, Co, and Mn) have been widely used as electrodes for electrocatalytic oxidation of HMF and showed high FDCA yield and Faradaic efficiency in recent years (Taitt et al., 2018; Cai et al., 2020; Bai et al., 2021; Zhang et al., 2021; Jing et al., 2023; Li et al., 2023; Lie et al., 2023). Ni-based materials show extremely high catalytic activity, which far exceeds that of other transition metals or metal alloys (Lu et al., 2020; Zhou et al., 2021b; Gao et al., 2022; Wu et al., 2022; Xu et al., 2022).The main reason is that Ni element has the abundant three-dimensional electron number and the unique, e.g., orbitals, which could enhance the covalency of the transition metal and oxygen bonds (Formenti et al., 2018; Dutta et al., 2019). Grabowski et al. (Grabowski et al., 1991)first reported the use of NiO/Ni(OH)_2_ for the electrocatalytic oxidation of HMF, and the FDCA yield was only 71%. Subsequently, various Ni-based compounds, such as nitrides (Zhang et al., 2019; Chen W. et al., 2021), phosphides (Lu et al., 2021), sulfides (Wang W. et al., 2021), borides (Barwe et al., 2018), oxides (Choi et al., 2020; Wang J. et al., 2021), hydroxides (Zhang et al., 2021), layered double hydroxides (LDHs) (Zhongming et al., 2021) and their composites, have been wildly investigated to electro-oxidation of HMF and demonstrated a high FDCA yield and FE. Despite these strategies developed for electrooxidation of HMF over nickel-based catalysts, designing of advanced materials and achieving high current densities at low potentials remains challenging.

The application of biomass-based material from renewable natural compounds is of great significance since the diversity of natural compounds provides unlimited possibilities and potential for designing abundant functional materials (Vinod et al., 2020; Zhang et al., 2022). Phytic acid (C_6_H_18_O_24_P_6_), for example, as a natural organic macromolecule distilled from grain, can coordinate with multiple metal ions and form strong bonds due to the presence of six phosphate groups and twelve hydroxyl groups in its structure (He et al., 2022). At that point, functional materials derived from phytic acid have shown great advantages and promising prospects in many fields, including coatings (Li et al., 2022; Song et al., 2022), catalysis (Wang et al., 2018; Gwóźdź and Brzęczek-Szafran, 2022; Song et al., 2023; Yang et al., 2023), and pharmaceuticals (Wang Y. et al., 2021). Herein, we prepared metal-phytic acid hybrids using natural phytic acid as building block, and Ni-phytic acid hybrid (Ni-PA) shows high catalytic performance in the electrochemical oxidation of HMF. The active Ni ion chelated with phytic acid to form nanomaterial with pore structure. It has high electrochemical active surface area and low charge transfer resistance. As a result, the Ni-PA catalyst exhibits outstanding performance for the oxidation of HMF to FDCA with high yield of 99.1% and FE for FDCA (90%) at 1.6 V vs. RHE.

## 2 Experimental section

### 2.1 Materials and methods

Anhydrous NiCl_2_ was purchased from Thermo Fisher Scientific, Anhydrous FeCl_3_, Anhydrous CuCl_2_, 5-hydroxymethylfurfural (HMF) and sodium phytate were purchased from Adamas, Nafion D-521 dispersion, Toray Carbon Paper (CP, TGP-H-60, 19 × 19 cm), Nafion N-117 membrane were purchased from Alfa Aesar China Co., Ltd. potassium hydroxide and potassium chloride were purchased from General-reagent. All reagents from commercial sources were used without further purification.

### 2.2 Synthesis of metal-PA

Synthesis of Ni-PA: In a typical synthesis, sodium phytate (5 mmol) and NiCl_2_ (15 mmol) were dissolved in deionized water (300 mL). The mixture was stirred at room temperature for 2 h and then aged in static conditions at room temperature for 12 h. Finally, the light green precipitate was separated by centrifugation and dried in the oven at 50 °C for 12 h.

Synthesis of Fe-PA and Cu-PA: The synthetic process of Fe-PA and Cu-PA is similar to Ni-PA except that the precursor NiCl_2_ is replaced by 15 mmol FeCl_3_ or CuCl_2_, respectively.

### 2.3 Catalyst characterization

The scanning electron microscopy (SEM) measurements were performed using a Hitachi S-4800 scanning electron microscope operated at 15 kV. Transmission electron microscopy (TEM) images were obtained from a JEOL-1011 resolution transmission electron microscopy. Powder X-ray diffraction (XRD) patterns were obtained on the X-ray diffractometer (Model D/MAX2500, Rigaka) with Cu-Kα radiation. X-ray photoelectron spectroscopy (XPS) analysis was performed on the Thermo Scientific ESCA Lab 250Xi using 200 W monochromatic Al Kα radiation, and the 500 μm X-ray spot was used. The base pressure in the analysis chamber was about 3 × 10^−10^ mbar. Typically, the hydrocarbon C1s line at 284.8 eV from adventitious carbon was used for energy referencing.

### 2.4 Electrochemical experiment

**Preparation of electrode:** 5 mg of the as-prepared materials and 10 μL of Nafion D-521 dispersion were dispersed in 1 mL absolute ethyl alcohol to create a homogeneous suspension by sonication. The suspension was then dripped on carbon paper (CP, 1 cm × cm). After drying in air, a catalyst with the loading of 5 mg cm^-2^ was obtained based on the weight change of the CP.

**Linear sweep voltammetry (LSV) measurements:** LSV measurements were performed using an electrochemical workstation (CHI 760E, Shanghai Chenhua Instrument Co., Ltd.). And typical H-type electrolytic cell was used, which is separated by a Nafion N-117 membrane. The H-type electrolysis cell contained three electrodes, including a working electrode, a platinum grid counter electrode, and an Ag/AgCl reference electrode. In addition, the anolyte electrolyte and catholyte electrolyte were 1 M KOH aqueous solution. LSV measurements were carried out under mild magnetic stirring, with a potential range of 1.0–2.0 V vs. RHE and a scanning speed of 10 mV/s.

**Electrochemical oxidation of HMF:** Electrochemical performance of the materials was assessed with a CHI 760 electrochemical workstation using a typical H-type cell at room temperature. The H-type electrolytic cell was divided into anode and cathode chambers with a Nafion N-117 proton exchange membrane. By the calculation of 0.197 + 0.059× pH, all potentials were converted from vs. Ag/AgCl to vs. RHE. The electrochemical oxidation experiment was performed in a 1.0 M KOH solution of 15 mL with or without 10 mM HMF.

## 3 Results and discussion

**FIGURE 1:**
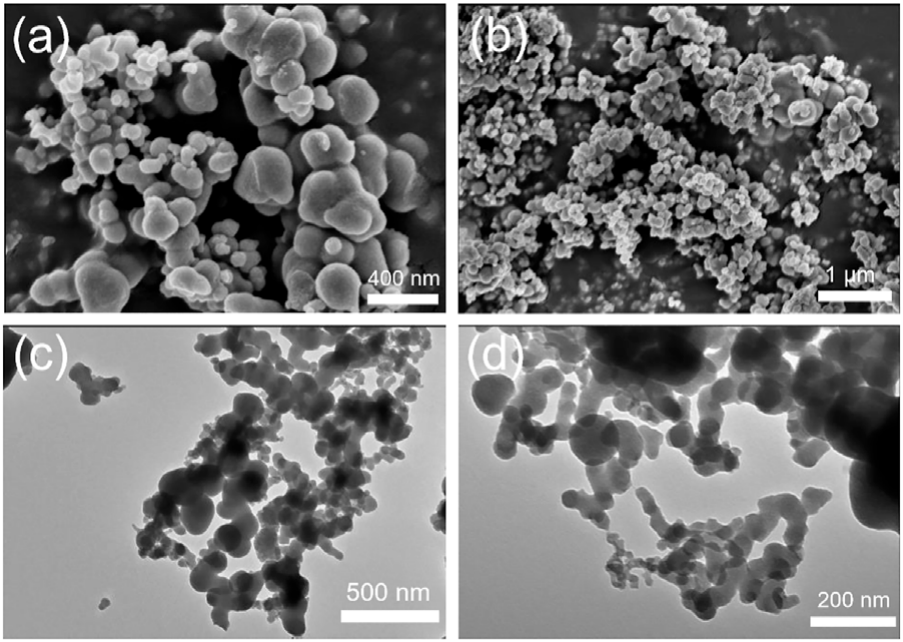
Characterizations of the prepared Ni-PA. **(A) (B)** SEM images, **(C) (D)** TEM images.

The prepared metal-phytic acid hybrids were first characterized by scanning electron microscopy (SEM) and transmission electron microscopy (TEM), and the morphology results are shown in Figure 1, Supplementary Figure S2 and S3. The SEM and TEM images of Ni-PA show that it has an irregular tubular and spherical shape (Figure 1). The diameter of the sphere is about 100–200 nm, and the length of irregular strip is between 200 and 300 nm with the width of about 100 nm. The Cu-PA catalyst shows similar morphology as Ni-PA, while the deformation of the tube is more serious (Supplementary Figure S1). And the Fe-PA consists of small particles with the diameter of about 40 nm (Supplementary Figure S2).

**FIGURE 2:**
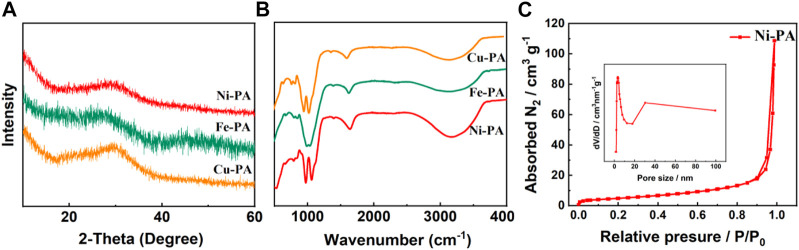
**(A)** XRD, **(B)** FTIR patterns of metal-phytic acid hybrids, **(C)** N_2_ adsorption–desorption isotherm and pore size distribution of Ni-PA.

The XRD patterns of various metal-phytic acid hybrids were shown in Figure 2A, which all exhibited the same structure characteristics. The results showed that all the metal-phytic acid hybrids were poorly ordered and amorphous, indicating the nanoparticles in metal-phytic acid hybrids are arranged irregularly, which is consistent with the SEM results (Song et al., 2015). Flourier transform infrared spectroscopy (FTIR) spectroscopy was then performed to research the chemical structures of metal-phytic acid hybrids samples (Figure 2B), and the peaks at 3400 cm^-1^ and 1642 cm^-1^ are corresponded to the absorbed H_2_O. The peak at 1063 cm^-1^ could be assigned to the Ni-O-P stretching vibrations, which clearly demonstrates the formation of coordination bond of Ni^2+^ ions and phosphate ester group (Xue et al., 2016; Xie et al., 2020). N_2_ adsorption–desorption isotherm and pore size distribution of Ni-PA in Figure 2C show that the catalyst has a typical microporous structure. The surface area and pore diameter are 64.1 m^2^/g and 0.71 nm, respectively. Cu-PA shows similar N_2_ adsorption–desorption isotherm with that of Ni-PA, while Fe-PA shows a typical mesoporous structure with the surface area of 125.8 m^2^/g and a pore diameter of 17.4 nm (Supplementary Figure S3 and Supplementary Table S1).

**FIGURE 3:**
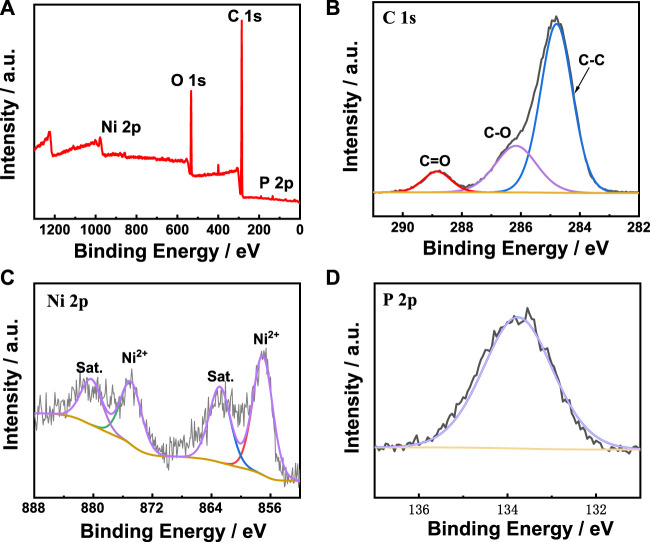
XPS spectra of Ni-PA catalyst. **(A)** full-scan spectrum, **(B)** C 1s, **(C)** Ni 2p, and **(D)** P 2p spectra.

2Moreover, the X-ray photoelectron spectroscopy (XPS) was carried out to analyze the electronic states and element composition of metal-phytic acid hybrids. The XPS spectrum of Ni-PA in Figure 3A shows that Ni-PA is mainly composed of C, O, P and Ni. For C 1s spectrum of Ni-PA, the peak can be fitted into three peaks at 284.8, 286.2 and 288.8 eV, corresponding to C-C, C-O and C=O (Figure 3B). The Ni 2p XPS spectrum of Ni-PA in Figure 3C shows the Ni 2p_1/2_ and Ni 2p_3/2_ binding energies at 874.7 and 857.0 eV, respectively, indicating the existence of Ni^2+^ in Ni-PA. The peak of P 2p (Figure 3D) at 133.8 eV corresponds to phytic acid. For O 1s (Supplementary Figure S4), two peaks at 532.1 eV and 533.5 eV could be obtained by peak split, and which corresponds to the C-O and C=O. The XPS spectra of Cu-PA and Fe-PA catalysts in Supplementary Figure S5 demonstrate that the catalysts consist of C, O, P, Cu and C, O, P, Fe, respectively. These results indicate that Ni/Cu/Fe element has been successfully coordinated with phytic acid.

**FIGURE 4:**
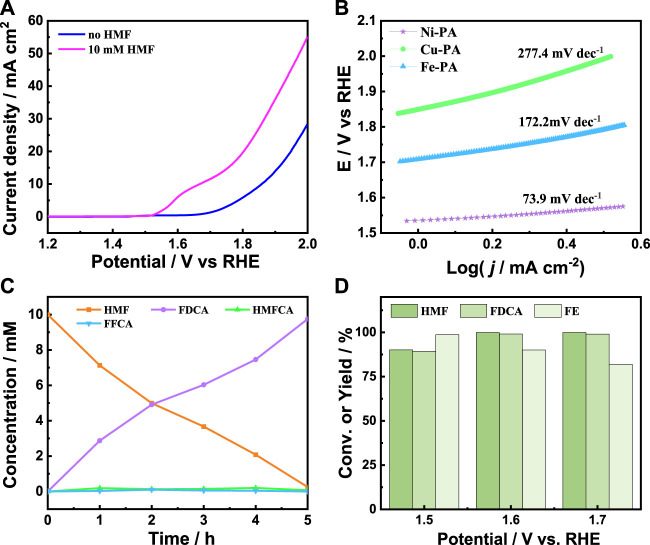
**(A)** LSV curves of Ni-PA with and without HMF at a scan rate of 0.1 V/s in 1.0 M aqueous KOH solution, **(B)** Tafel plots of metal-phytic acid hybrids with HMF, **(C)** Concentrations of HMF and the oxidation products during electrolysis, and **(D)** The HMF conversion, yield and FE of FDCA at different applied potentials over Ni-PA.

The electrocatalytic performance of HMF oxidation over metal-phytic acid hybrids was firstly investigated by linear sweep voltammetry (LSV) in 1.0 M KOH electrolyte. Figure 4A present the LSV curves of Ni-PA with and without 10 mM HMF, respectively, and the oxidation peak at 1.67 V is associated with the oxidation of Ni^2+^ to higher oxidation state (Xu et al., 2022). The onset potential shifted from 1.64 V to 1.50 V after adding 10 mM HMF, and the current density increased dramatically with 10 mM HMF, indicating that the oxidation of HMF was more favorable than water oxidation. Besides, as shown in Figure 4A and Supplementary Figure S6, Ni-PA possessed the highest current density and a lowest current density onset potential among the metal-phytic acid hybrids, indicating Ni-PA was more active to the HMF oxidation than Cu-PA and Fe-PA. The Tafel slope of Ni-PA with HMF was calculated as 73.9 mV dec^-1^ (Figure 4B), much lower than that of Cu-PA (277.4 mV dec^-1^) and Fe-PA (172.2 mV dec^-1^), indicating a much faster catalytic kinetics of Ni-PA at the anode after adding HMF. Based on these results, the constant potential electrolysis of HMF was conducted over Ni-PA in 1.0 M KOH electrolyte at an applied potential of 1.6 V vs. RHE. High-performance liquid chromatography (HPLC) was used to obtain the concentration of HMF and oxidation products. As can be seen from Figure 4C, the concentration of HMF decreased and the amount of FDCA increased with the extension of reaction time, indicating that HMF was successfully oxidized to FDCA. In addition, there is a small amount of 5-hydroxymethyl-2-furanocarboxylic acid (HMFCA) and 2-formyl-5-furanocarboxylic acid (FFCA) could be detected during the reaction time, which have been reported as common reaction intermediates in the electrolysis of HMF to FDCA (Fu et al., 2022). After 5 h electrolysis at 1.6 V, HMF could be completely converted to FDCA, and the yield and Faraday efficiency of FDCA could reach 99.1% and 90%, respectively. Moreover, Ni-PA exhibited better HMFOR activity compared to other reported electrocatalysts (Supplementary Table S2). The applied potential also affects the HMF oxidation results. As shown in Figure 4D, the yield of FDCA increases with the increase of applied potential, while the Faraday efficiency decreases with the increase of applied potential. Considering both the FDCA yield and Faraday efficiency of the reaction, the most suitable potential in our catalytic system is 1.6 V vs. RHE. In addition, Ni-PA showed a superior yield of FDCA compared with Cu-PA and Fe-PA (Supplementary Figure S7), indicating the superior activity of Ni-PA for HMF oxidation to FDCA. The durability test of Ni-PA was then conducted through five electrolysis. As shown in Supplementary Figure S8, there was no significant decrease in the yield and FE of FDCA. The Ni-PA recovered after five times reuses was then characterized by XPS, FT-IR, XRD, SEM and TEM (Supplementary Figure S9, S10), and no notable difference could be found, indicating the good cyclic stability.

**FIGURE 5:**
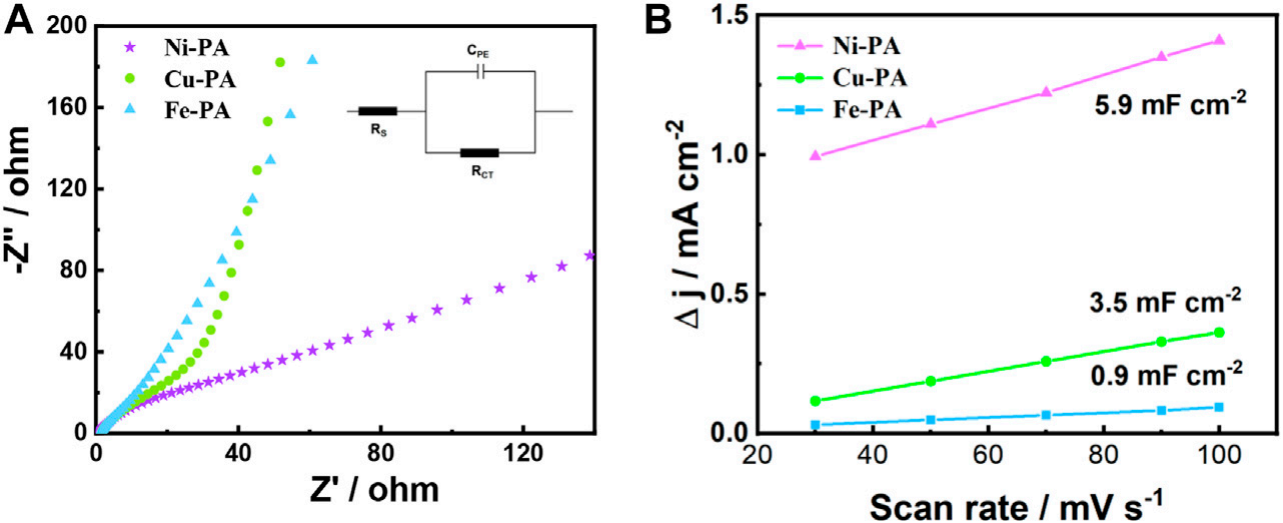
**(A)** Nyquist plots (inside is the equivalent circuit of electrochemical impedance spectroscopy), and **(B)** Charging current density differences plotted against scan rates of various metal-phytic acid electrodes in 1.0 M KOH solution.

To investigate the reason for the excellent performance of the Ni-PA on the electro-oxidation of HMF to FDCA, electrochemical impedance spectroscopy (EIS) and cyclic voltammetry (CV) measurements were performed to investigate the electrode/electrolyte interface properties of various metal-phytic acid electrodes. The Nyquist plots of various metal-phytic acid electrodes were shown in Figure 5A. In which Ni-PA performed the lowest charge transfer resistance (Rct) of 1.8 Ω, much smaller than that of Cu-PA (3.9 Ω) and Fe-PA (4.2 Ω), respectively, indicating a more facile electron transfer process and faster reaction rate. Besides, the electrochemical surface area (ECSA) results obtained from the double-layer capacitance in CV curves against scan rates (Supplementary Figure S11) are shown in Figure 5B. It is obvious that Ni-PA possess the maximum slope of 5.9 mF cm^-2^ among various metal-phytic acid electrodes, thus has a highest electrochemical active surface area. It indicates that there are more reactive sites in Ni-PA for HMF, probably driving from the coordination of Ni and phytic acid.

## 4 Conclusion

In summary, various metal-phytic acid hybrids (Ni-PA, Fe-PA, Cu-PA) have been successfully synthesized by precipitation method. The prepared catalysts were investigated to electrochemical oxidation of HMF and Ni-PA showed excellent activity to produce FDCA. The yield and Faradaic efficiency of FDCA could reach 99.1% and 90% respectively at an applied potential of 1.6 V vs. RHE, much higher than that of Fe-PA and Cu-PA. Electrochemical tests show that Ni-PA has high electrochemical surface area and low charge transfer resistance, which enhanced the activity and selectivity for the electrochemical oxidation of HMF to FDCA. We believe that this work provides an efficient strategy to explore the biomass resources to prepare functional materials, which may have great application in the field of catalysis.

## Data Availability

The original contributions presented in the study are included in the article/Supplementary Material, further inquiries can be directed to the corresponding author.
