# Supplementary material for: Nickel-phytic acid hybrid for highly efficient electrocatalytic upgrading of HMF
Source: Front Chem. 2023 May 18;11:1199921. doi: 10.3389/fchem.2023.1199921 (PMC10232861; doi:10.3389/fchem.2023.1199921)
Supplement: Supplementary file 1 [file DataSheet1.docx]

Supplementary Material

Nickel-phytic acid hybrid for highly efficient electrocatalytic upgrading of HMF

Shuyi Liu^†^, Xue Yuan^†^, Xin Huang^*^, Yu Huang, Chen Sun, Kun Qian, Wenjie Zhang

*** Correspondence:** Xin Huang: huangxin@cugb.edu.cn


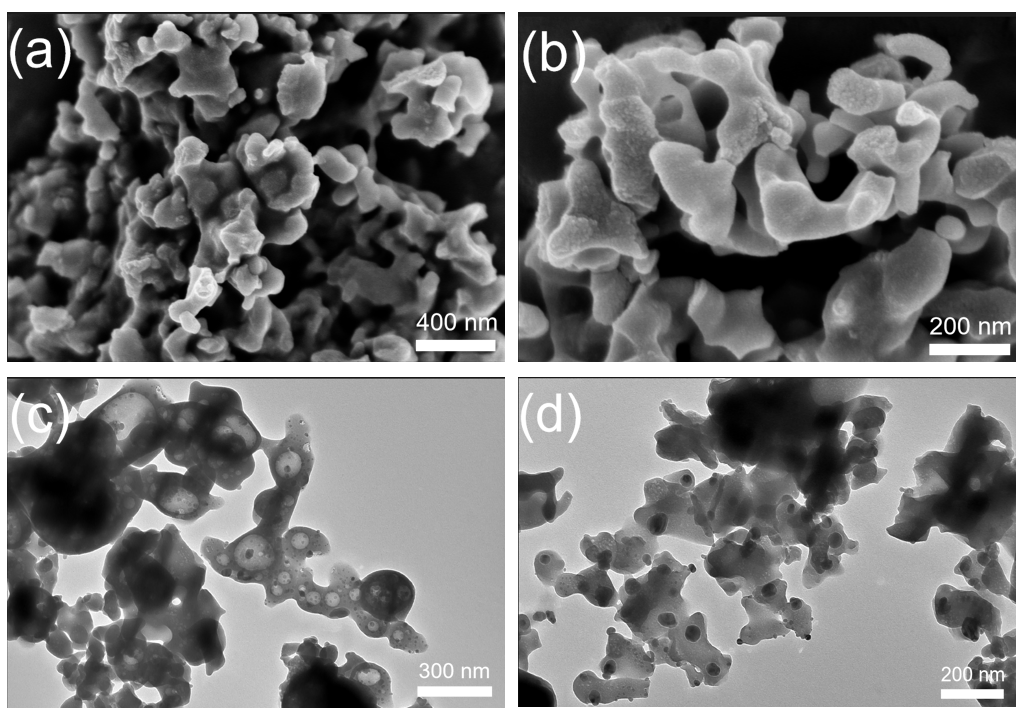


**Supplementary Figure 1.** Characterizations of the prepared Cu-PA. (a) (b) SEM images, (c) (d) TEM images.

**Supplementary Figure 2.** Characterizations of the prepared Fe-PA. (a) (b) SEM images, (c) (d) TEM images.


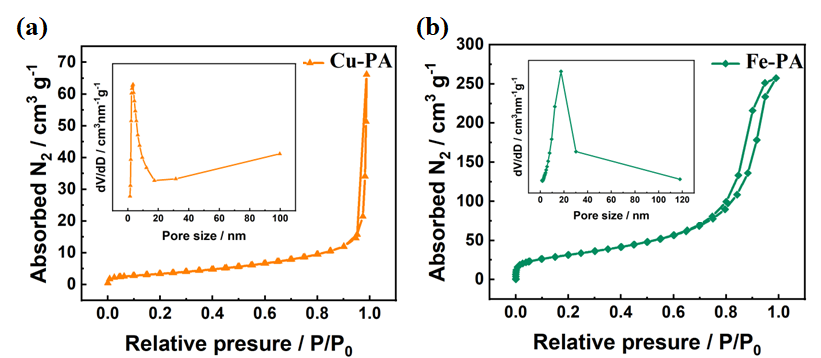


**Supplementary Figure 3.** N_2_ adsorption–desorption isotherm and pore size distribution of Cu-PA and Fe-PA.

**Supplementary Figure 4.** O 1s spectra of Ni-PA.


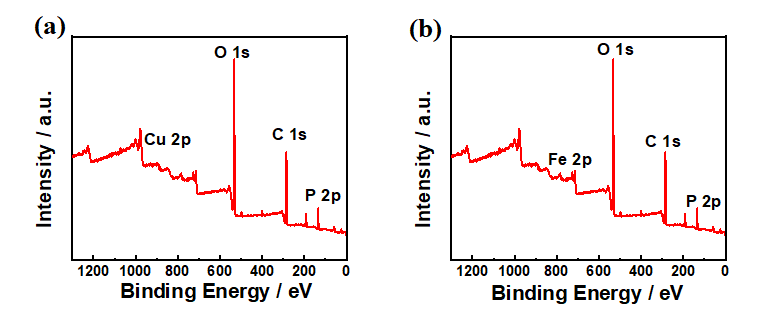


**Supplementary Figure 5.** XPS spectra of Cu-PA and Fe-PA catalysts.


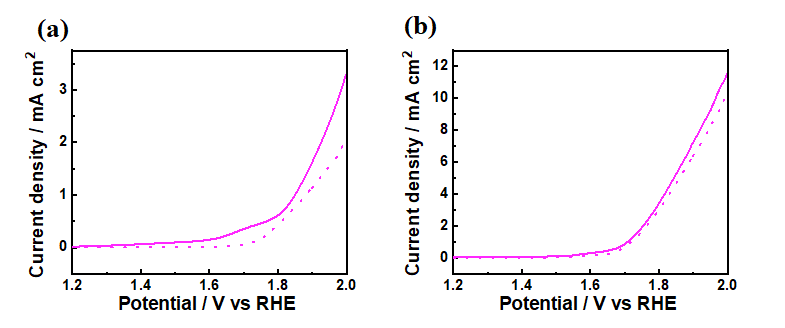


**Supplementary Figure 6.** LSV curves of Ni-PA at a scan rate of 0.1 V/s in 1.0 M aqueous KOH solution of (a) Cu-PA and (b) Fe-PA.

**Supplementary Figure 7.** HMF conversion and yield of FDCA over various metal-phytic acid hybrids at 1.6 V *vs*. RHE.

**Supplementary Figure 8.** Reusability of Ni-PA after reused for five times.


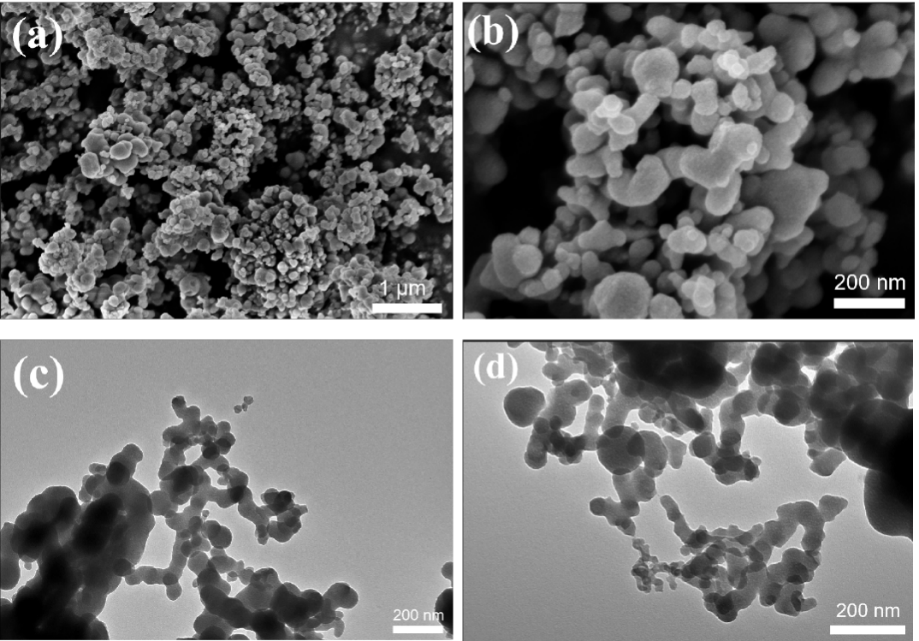


**Supplementary Figure 9. (a) (b)** SEM image and (c) (d) TEM image of Ni-PA after reused for five times.

**
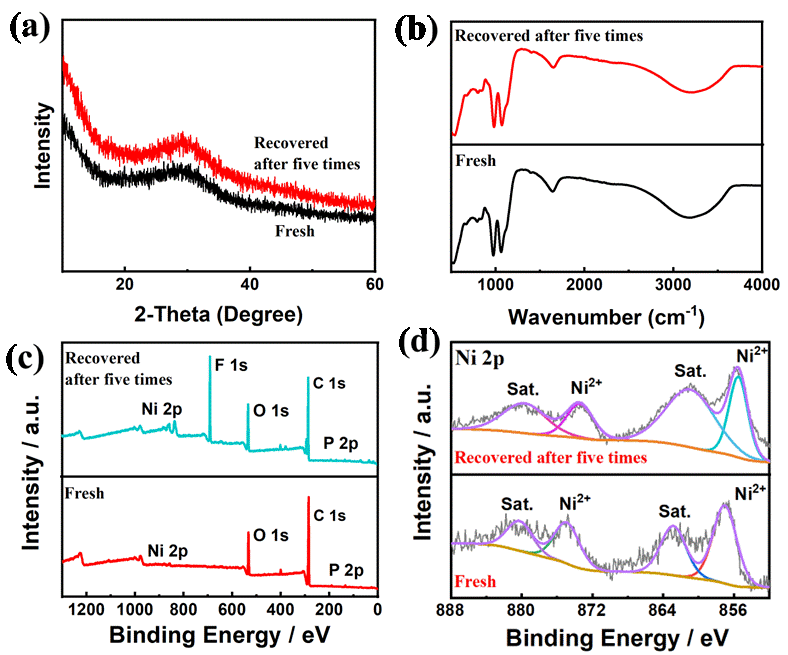
**

**Supplementary Figure 10.** The characterization of Ni-PA catalyst after reused for five times. XRD pattern (a), FT-IR spectrum (b). XPS spectra of Ni-PA catalyst. full-scan spectrum(c), Ni 2p spectra (d). The XRD spectra and FTIR spectra demonstrate that the structure of Ni-PA remains stable. In the XPS spectrum, the Ni-PA of recovered after five times showed a new peak at ~688 cm^-1^, corresponding to the F1s peak, probably due to the addition of Nafion-D521 during the preparation of the Ni-PA catalyst.

**
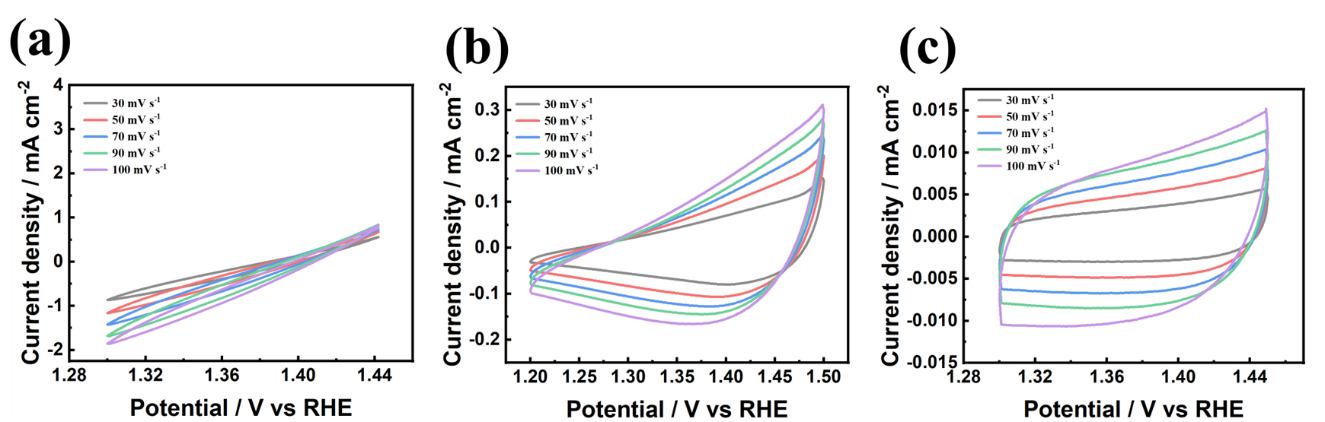
**

**Supplementary Figure 11.** CV curves of Ni-PA(a),Cu-PA(b) and Fe-PA(c) at different scan rates.

**Table S1.** Properties of metal-phytic acid hybrids.

| Sample | BET Surface Area  (m^2^ g^-1^) | Pore Diameter  (nm) | Pore volume  (cm^3^ /g) |
| --- | --- | --- | --- |
| Ni-PA | 64.1 | 0.71 | 0.18 |
| Cu-PA | 39.9 | 0.74 | 0.11 |
| Fe-PA | 125.8 | 17.4 | 0.40 |

Table S2. Comparison HMFOR activity of Ni-PA with other electrocatalysts.

| **Samples** | **Electrolyte** | **Oxidation**  **voltage**  **(V vs RHE)** | **HMF Conversion (%)** | **FDCA yield**  **(%)** | **Faraday efficiency (%)** | **Reference** |
| --- | --- | --- | --- | --- | --- | --- |
| Ni-PA | 1.0 M KOH | 1.6 | 100 | 99.1 | 90 | This work |
| NiO-N/C | 1.0 M KOH | 1.473 | 99 | 84 | 96 | (Wang et al., 2022) |
| WO_3_/ Ni | 1.0 M KOH | 1.44 | 99.4 | 88.3 | 88.0 | (Hu et al., 2021) |
| Ce–CoP | 1.0 M KOH | 1.45 | 100 | 98 | 96.4 | (Bi et al., 2022) |
| Ni_2_S_3_/NF | 1.0 M KOH | 1.498 | 100 | 98 | 94 | (Wang et al., 2021b) |
| N-NiMoO_4_/NF | 1.0 M KOH | 1.473 | 100 | 97 | 91 | (Wang and Wang, 2021) |
| TpBpy-Ni@FTO | 0.1 M LiClO_4_ | 1.55 | 96 | 58 | ___ | (Cai et al., 2020) |
| Co(OH)_2_@ZIF-67 | 1.0 M KOH | 1.42 | 90.9 | 81.8 | 83.6 | (Pila et al., 2021) |
| Ni/Ni_0.2_Mo_0.8_N/NF | 1 M KOH | 1.51 | 100 | 98.5 | 99.3 | (Sun et al., 2023) |
| NiB_x_-P_0.07_ (nP/nNi=0.07) | 1 M KOH | 1.464 | 99 | 90.6 | 92.5 | (Song et al., 2020) |
| RuO_2_/MnO_2_/CNT | 0.1 M K_2_SO_4_ | 0.9 | ___ | 72.1 | ~100 | (Wang et al., 2021a) |

**References**

Bi, J., Ying, H., Xu, H., Zhao, X., Du, X., Hao, J., et al. (2022). Phosphorus vacancy-engineered Ce-doped CoP nanosheets for the electrocatalytic oxidation of 5-hydroxymethylfurfural. *Commun. Chem.*58**,** 7817-7820. <https://doi.org/10.1039/D2CC02451A>

Cai, M., Ding, S., Gibbons, B., Yang, X., Kessinger, M.C., and Morris, A.J. (2020). Nickel (ii)-modified covalent-organic framework film for electrocatalytic oxidation of 5-hydroxymethylfurfural (HMF). *Commun. Chem.* 56**,** 14361-14364. <https://doi.org/10.1039/D0CC02206C>

Hu, K., Zhang, M., Liu, B., Yang, Z., Li, R., and Yan, K. (2021). Efficient electrochemical oxidation of 5-hydroxymethylfurfural to 2, 5-furandicarboxylic acid using the facilely synthesized 3D porous WO3/Ni electrode. *Mol. Catal.* 504**,** 111459. <https://doi.org/10.1016/j.mcat.2021.111459>

Pila, T., Nueangnoraj, K., Ketrat, S., Somjit, V., and Kongpatpanich, K. (2021). Electrochemical Production of 2, 5-Furandicarboxylic from 5-Hydroxymethylfurfural Using Ultrathin Co (OH) 2 on ZIF-67. *ACS Appl. Energy Mater.* 4**,** 12909-12916. <https://doi.org/10.1021/acsaem.1c02543>

Song, X., Liu, X., Wang, H., Guo, Y., and Wang, Y. (2020). Improved performance of nickel boride by phosphorus doping as an efficient electrocatalyst for the oxidation of 5-hydroxymethylfurfural to 2, 5-furandicarboxylic acid. *ACS Appl. Energy Mater.* 59**,** 17348-17356. <https://doi.org/10.1021/acs.iecr.0c01312>

Sun, M., Yang, J., Huang, J., Wang, Y., Liu, X., Qi, Y., et al. (2023). Interfacial Engineering of Ni/Ni_0. 2_Mo_0. 8_N Heterostructured Nanorods Realizes Efficient 5-Hydroxymethylfurfural Electrooxidation and Hydrogen Evolution. *Langmuir*. <https://doi.org/10.1021/acs.langmuir.2c03494>

Wang, T., Song, Y., Zhao, W., Zhou, C., Jin, Y., Wan, X., et al. (2021a). Electro-catalytic oxidation of HMF to FDCA over RuO _2_/MnO _2_/CNT catalysts in base-free solution. *New J. Chem.* 45**,** 21285-21292. <https://doi.org/10.1039/D1NJ03292E>

Wang, W., Kong, F., Zhang, Z., Yang, L., and Wang, M. (2021b). Sulfidation of nickel foam with enhanced electrocatalytic oxidation of 5-hydroxymethylfurfural to 2, 5-furandicarboxylic acid. *Dalton Trans.* 50**,** 10922-10927. <https://doi.org/10.1039/D1DT02025K>

Wang, W., and Wang, M. (2021). Nitrogen modulated NiMoO 4 with enhanced activity for the electrochemical oxidation of 5-hydroxymethylfurfural to 2, 5-furandicarboxylic acid. *Catal. Sci. Technol.* 11**,** 7326-7330. https://doi.org/10.1039/D1CY00786F

Wang, W., Zhang, Z., and Wang, M. (2022). Preparation of NiO-N/C composites for electrochemical oxidation of 5-hydroxymethylfurfural to 2, 5-furandicarboxylic acid. *Biomass Convers. Biorefin.***,** 1-8. https://doi.org/10.1007/s13399-022-02949-5
